# Chronic heart failure modifies respiratory mechanics in rats: a randomized controlled trial

**DOI:** 10.1590/bjpt-rbf.2014.0163

**Published:** 2016-04-08

**Authors:** Deise M. Pacheco, Viviane D. Silveira, Alex Thomaz, Ramiro B. Nunes, Viviane R. Elsner, Pedro Dal Lago

**Affiliations:** 1 Laboratório de Fisiologia Cardiovascular, Universidade Federal de Ciências da Saúde de Porto Alegre (UFCSPA), Porto Alegre, RS, Brazil; 2 Programa de Pós-graduação em Ciências da Saúde, UFCSPA, Porto Alegre, RS, Brazil; 3 Programa de Pós-graduação em Ciências da Reabilitação, UFCSPA, Porto Alegre, RS, Brazil; 4 Programa de Pós-graduação em Biociências e Reabilitação, Centro Universitário Metodista do IPA, Porto Alegre, RS, Brazil

**Keywords:** heart failure, respiratory mechanics, controlled trial, pulmonary edema, cardiac hypertrophy, rats

## Abstract

**Objective:**

To analyze respiratory mechanics and hemodynamic alterations in an experimental model of chronic heart failure (CHF) following myocardial infarction.

**Method:**

Twenty-seven male adult Wistar rats were randomized to CHF group (n=12) or Sham group (n=15). Ten weeks after coronary ligation or sham surgery, the animals were anesthetized and submitted to respiratory mechanics and hemodynamic measurements. Pulmonary edema as well as cardiac remodeling were measured.

**Results:**

The CHF rats showed pulmonary edema 26% higher than the Sham group. The respiratory system compliance (Crs) and the total lung capacity (TLC) were lower (40% and 27%, respectively) in the CHF rats when compared to the Sham group (*P<0.01)*. There was also an increase in tissue resistance (Gti) and elastance (Hti) (28% and 45%, respectively) in the CHF group. Moreover, left ventricular end-diastolic pressure was higher (32 mmHg vs 4 mmHg, *P<0.01)*, while the left ventricular systolic pressure was lower (118 mmHg vs 130 mmHg, *P*=0.02) in the CHF group when compared to the control. Pearson’s correlation coefficient showed a negative association between pulmonary edema and Crs (*r*=–0.70, *P*=0.0001) and between pulmonary edema and TLC (*r*=–0.67, *P*=0.0034). Pulmonary edema correlated positively with Gti (*r*=0.68, *P*=0.001) and Hti (*r*=0.68, *P*=0.001). Finally, there was a strong positive relationship between pulmonary edema and heart weight (*r*=0.80, *P*=0.001).

**Conclusion:**

Rats with CHF present important changes in hemodynamic and respiratory mechanics, which may be associated with alterations in cardiopulmonary interactions.

## Bullet points

- Chronic heart failure (CHF) modifies mechanical respiratory and hemodynamic parameters.
- CHF induces pulmonary congestion.
- CHF increases tissue resistance and elastance.
- CHF increases left ventricular end-diastolic pressure.

## Introduction

Chronic heart failure (CHF) is a complex syndrome described by the inability of cardiac muscle to maintain peripheral tissue blood supply, which may impair the energetic metabolism of the whole body^1^. The most common clinical symptoms observed in CHF following myocardial infarction (MI) are dyspnea, muscle weakness, and early fatigue. These factors have been associated with functional limitation, which contributes to reduced exercise tolerance and poor health status and quality of life^1^ ^,^ ^2^.

Left ventricular (LV) remodeling following MI is a dynamic and complex process that occurs in response to myocardial damage. Initially, LV dilatation is considered a protective mechanism to maintain cardiac systolic function. However, this phenomenon ultimately leads to alterations in LV global function and aggravates heart failure. Additionally, progressive LV dilatation after MI has been documented as a strong predictor of both CHF and death^3^.

It is noteworthy that the cardiovascular system is intimately linked to the respiratory system. Mechanistic studies have shown that dyspnea in CHF results from increased airway resistance and reduced pulmonary compliance^4^. Furthermore, the breathing reserve in CHF is reduced as a result of increased respiratory system (Rrs) resistance. Importantly, increased Rrs contributes to expiratory flow limitation and limited response in minute ventilation (VE) during hypercapnia^5^ ^-^ ^7^. Significant alterations in respiratory system compliance (Crs) and reduced endurance of the inspiratory muscles are also observed in CHF^8^ ^-^ ^10^, which have been linked to changes in the total lung capacity (TLC). In this context, it has been demonstrated that lung congestion in pulmonary edema models immediately after MI induces a progressive reduction in respiratory system compliance^11^ ^-^ ^13^. Despite this evidence, studies regarding cardiopulmonary interactions in CHF are scarce, and most of the available findings are focused on the individual contributions of the respiratory and cardiovascular systems, without describing the relationship between them. Besides, the experimental studies reporting respiratory and cardiovascular alterations address only the acute effects of CHF^14^, and less attention has been paid to the chronic outcomes. Hence, the aim of the present study was to evaluate the effect of a CHF model following MI on pulmonary and cardiovascular parameters and their interactions in the CHF rats.

## Method

### Experimental design

Twenty-seven adult male Wistar rats (body weight 190 to 230 g) were obtained from the Animal Breeding Unit of Universidade Federal de Ciências da Saúde de Porto Alegre (UFCSPA), Porto Alegre, RS, Brazil. With randomly permuted blocks, the rats were randomly allocated to chronic heart failure group (CHF, *n*=12) or to sham surgery group (Sham, *n*=15) in blocks to ensure that an equal number of rats had been assigned to each group each time using a randomization website. The animals were housed three per cage, receiving food and water *ad libitum* in an animal room under a 12-hour light-dark cycle, at 22 °C.

The investigation followed the ethical rules established by the Guide for the Care and Use of Experimental Animals published by the National Institute of Health (NIH publication 85-23, revised in 1996). All procedures outlined in this study were approved by the Research Ethics Committee of the UFCSPA (protocol 127/05).

### Surgery to Induce Myocardial Infarction (MI)

The rats were anesthetized with xylazine (12 mg/Kg, i.p.) and ketamine (90 mg/Kg, i.p.), intubated and artificially ventilated (SamWay VR 15) with a respiratory frequency of 60 breaths per minute and a fraction of inspired oxygen of 100%. MI was induced as previously described by Pfeffer et al.^15^. The heart was exposed through a left thoracotomy between the fourth and fifth ribs. For the animals in which MI was induced, a 6-0 mononylon suture was passed around the main left descending coronary artery at a point between 1 and 2 mm distal to the edge of the left atrium, and a coronary ligature was performed.

Sham-operated animals underwent the same procedure without tying the suture. The thorax was closed, the skin was sutured and the pneumothorax was drained using a continuous aspiration system. The rats received a single dose of penicillin (20,000 IU, i.p.) and naproxen (40 mg/Kg, i.m.) and were then placed in a heated environment for recovery. After the surgery, the rats remained in their cages for a period of 10 weeks. All animals were weighed at the beginning and the end of the experiment.

### Measurements

#### Assessment of respiratory mechanics

Ten weeks after MI or sham surgery, the rats were anesthetized again and tracheotomized, with a rigid cannula (2-mm ID) being inserted into the trachea and firmly tied in place. The cannula was connected to a small animal ventilator (flexiVent, SCIREQ Inc., Montreal, PQ, Canada). Rats were mechanically ventilated at a frequency of 90 breaths/min, with a tidal volume (VT) of 10 mL/Kg, using a 5-cmH_2_O level of positive end-expiratory pressure (PEEP) that was established using a water trap. While no muscle relaxants were used, if necessary, additional doses of anesthetics were given as required. In addition, the respiratory rate was set above normal (120 breaths/min) to suppress spontaneous breathing when measuring respiratory mechanics^16^. The animals were allowed to stabilize on the ventilator for 5 min before the measurements were taken.

The 16-s volume perturbation signal was used for the respiratory system impedance (Zrs) measurement. Prior to experiments with each rat, calibration signals were collected by applying the volume oscillation through the tracheal cannula, first with the cannula completely closed and then open to the atmosphere, as described previously^17^. The perturbation of 16 s is composed of sinusoids with mutually prime frequencies ranging from 0.25 to 19.625 Hz, which were chosen to avoid harmonic distortion^18^. The amplitudes of the sinusoids decreased hyperbolically with frequency. Calculation of the data corrections were made, considering the losses due to the compressibility of gases^19^.

Piston volume displacement (V*_cyl_*) was corrected to identify the true volume (V) that reached the animal, and cylinder pressure (P*_cyl_*) was corrected in order to obtain the airway opening pressure value (P*_ao_*). Flow (V’) was determined using the time derivation of V.

The constant-phase model described by Hantos et al.^20^ was used to partition Zrs into components representing the mechanical properties of the airway and parenchyma. The constant-phase model was fitted as follows: Zrs (*f*)= R*_aw_* + *i*2*πf* I*_aw_* + Gti – *iHti/(2π. f)^α^*, where R*_aw_* is airway resistance, I*_aw_* is the inertance, Gti is the coefficient of tissue damping that characterizes the dissipation of energy in the lung tissue, Hti is the co-efficient of tissue elastance that is characterized by the energy accumulated in the lung tissues, *i* is the imaginary unit, *f* is frequency, and α=(2/π).

Unlike the model that uses the equation of motion to obtain the data of resistance and elastance of the respiratory system, this constant-phase model considers the pulmonary system in different compartments of the lungs. The airway resistance (Raw) parameter allows analysis of the airway alone without interference from the lung tissue. The Gti evaluates the tissue resistance, whereas Hti evaluates the elastance of lung tissue^21^.

#### Cardiac hemodynamic evaluation

After evaluating the respiratory mechanics, while under anesthesia, as described previously, a polyethylene catheter (PE-50) was inserted into the right carotid artery for hemodynamic evaluation. The arterial pressure was first recorded during a 5-minute period by connecting the arterial cannula to a pressure transducer (Strain-Gauge - Narco Biosystem Miniature Pulse Transducer RP-155, Houston, TX, USA) coupled to a pressure amplifier (Stemtec). Next, the catheter was positioned within the left ventricle and the pulse wave was monitored using the typical graphic registration of ventricular pressure and was also recorded for 5 minutes. Analogical pressure signals were digitized by means of a data acquisition system (CODAS - Data Acquisition System) with a sampling rate of 2000 Hz. These data were used to determine mean arterial pressure (MAP), heart rate (HR), left ventricular systolic pressure (LVSP), left ventricular peak velocities of contraction (positive dP/dt_max_), left ventricular peak velocities of relaxation (negative dP/dt_max_), and left ventricular end-diastolic pressure (LVEDP).

#### Infarcted area, cardiac remodeling and pulmonary edema evaluation

After the procedures describe above, the animals were sacrificed with an overdose of anesthetic (thiopental 80 mg/Kg; i.p.). The heart and lungs were dissected and weighed. The heart was divided into the right ventricle (RV) and the left ventricle (LV).

The myocardial infarcted area was evaluated by planimetry^22^. To assess the development of cardiac remodeling, the combined ratios of RV and LV to body weight (HW/BW) were measured. In order to verify the magnitude of LV and RV remodeling, the LV-to-body weight (LV/BW) and RV-to-body weight (RV/BW) were also analyzed. Pulmonary edema was measured by the lung-to-body-weight (LW/BW) ratio as previously described^23^.

### Statistical analysis

Data are expressed as the mean ± standard deviation. The Kolmogorov–Smirnov test was performed to evaluate the normality of all variables. Inter-group data were compared using the Student t test for unpaired data. A *P* value of less than 0.05 was considered statistically significant. Linear regression analysis was used to examine the relationships between the values obtained for the respiratory mechanical parameters and lung edema (LW/BW ratio) and cardiac remodeling (HW/BW ratio) and the LW/BW ratio. Significance levels were determined using Pearson’s *r* correlation coefficient. GraphPad Prism 4 (GraphPad Software, San Diego, CA, USA) and SigmaPlot 11.0 (Systat Software Inc., San Jose, CA, USA) for Windows were used for data analysis.

## Results

### Mortality and body weight

The mortality in the MI-induced CHF rats during or immediately after surgery was 29.1%, while mortality during the 10-week of recovery period was 5.5%. In the Sham group, there were no deaths during the study. No significant differences in body weight were found between the groups in both moments of the study (Table 1).

**Table 1:** Changes in physiological parameters due to MI-induced CHF. Values are means±SD.

	**CHF (n=12)**	**Sham (n=15)**	**P Values**
Initial BW (g) Final BW (g) HR (bpm) MAP (mmHg) HW/BW (mg⁄g) LV/BW (mg⁄g) RV/BW (mg⁄g) LW/BW (mg⁄g)	214±4 306±7 269±29 114±5 4.24±0.15 3.28±0.14 1.05±0.14 2.48±0.16	219±3 322±7 295±23 129±4 3.37±0.07 2.84±0.08 0.54±0.03 1.84±0.05	0.34 0.13 0.49 0.03 0.0001 0.008 0.0008 0.0003

BW: body weight; HR: heart rate; MAP: mean arterial pressure; LV: left ventricle; RV: right ventricle.

### Infarcted area, pulmonary edema, and cardiac remodeling

At the end of the study, the infarcted area size was around 35%. The CHF group showed increased pulmonary edema compared to the Sham group (*P*=0.0003). Furthermore, the HW/BW ratio was higher in the CHF rats compared to the Sham group (*P*=0.0001), which is an indication of cardiac remodeling. Interestingly, the CHF group also showed an increase in both LV/BW and RV/BW ratios compared to the Sham group (*P*=0.008 and *P*=0.0008, respectively). All of these data are summarized in Table 1.

### Hemodynamic variables

**Figure 1:**
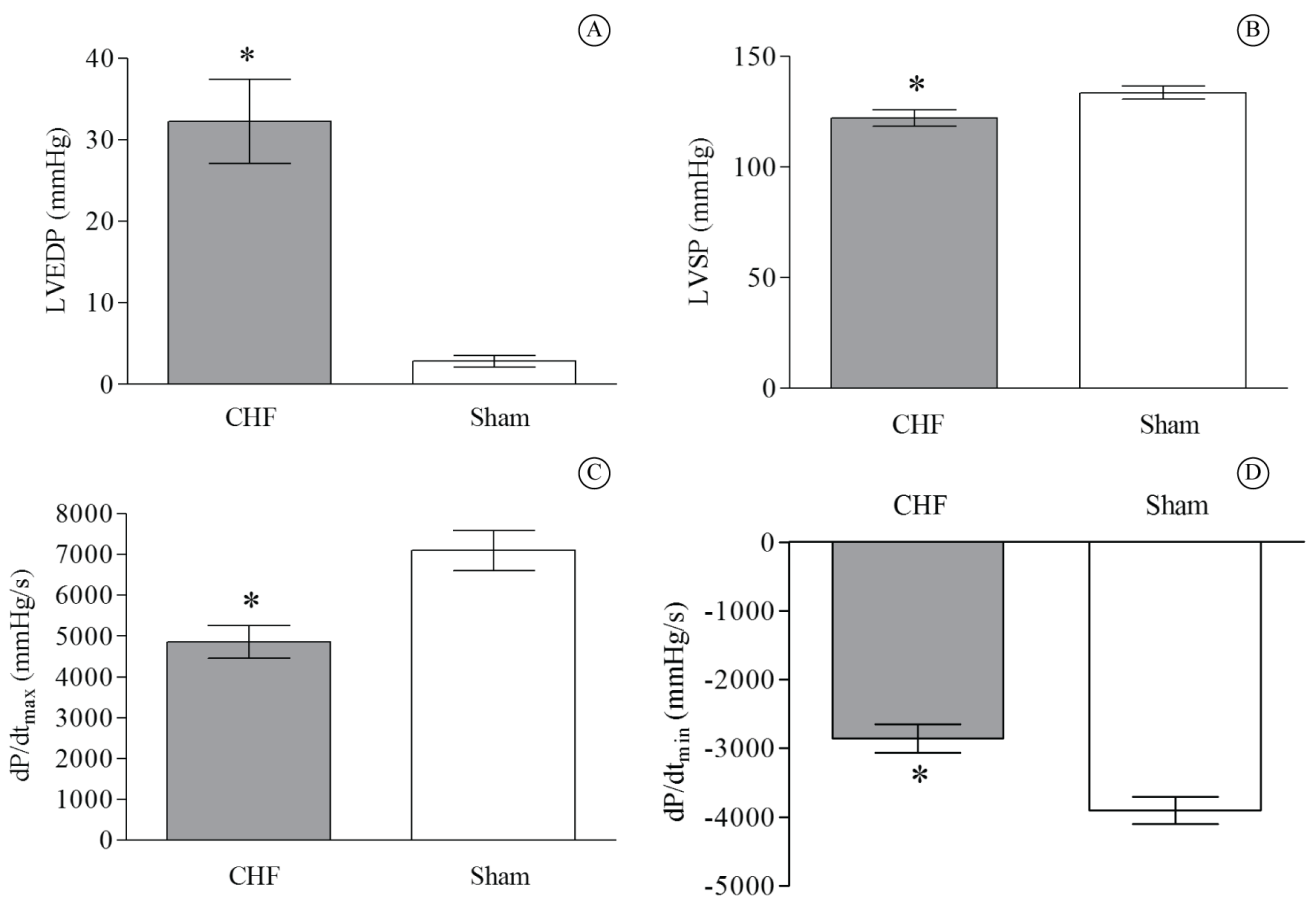
Hemodynamic characteristics of chronic heart failure (CHF) compared to sham-surgery group. CHF group (*n*=12) *vs* Sham group (*n*=15). (A) Left ventricular end-diastolic pressure (LVEDP) *P=0.001; (B) Left ventricular systolic pressure (LVSP) *P=0.0232; (C) Left ventricular maximum change in pressure over time (dP∕dt_max_) *P=0.005; (D) Left ventricular minimum change in pressure over time (dP∕dt_min_) *P=0.002. Values are means±SD.

When compared to the Sham group, the CHF animals presented a significant reduction in MAP (*P*=0.03), but no difference in HR was found between the groups (Table 1). Figure 1 highlights the differences in hemodynamic alterations between the CHF and Sham groups. It was observed that the CHF rats presented a higher LVEDP value compared to the Sham group (*P*=0.001; Figure 1A), while reduced LVSP values were found in the CHF group (p=0.023, Figure 1B). Finally, the CHF rats showed a reduction in positive dP/dt_max_ and negative dP/dt_max_ parameters when compared to the Sham group (*P*=0.005 and *P*=0.002, respectively; Figure 1C and 1D).

### Respiratory mechanics variables

**Figure 2:**
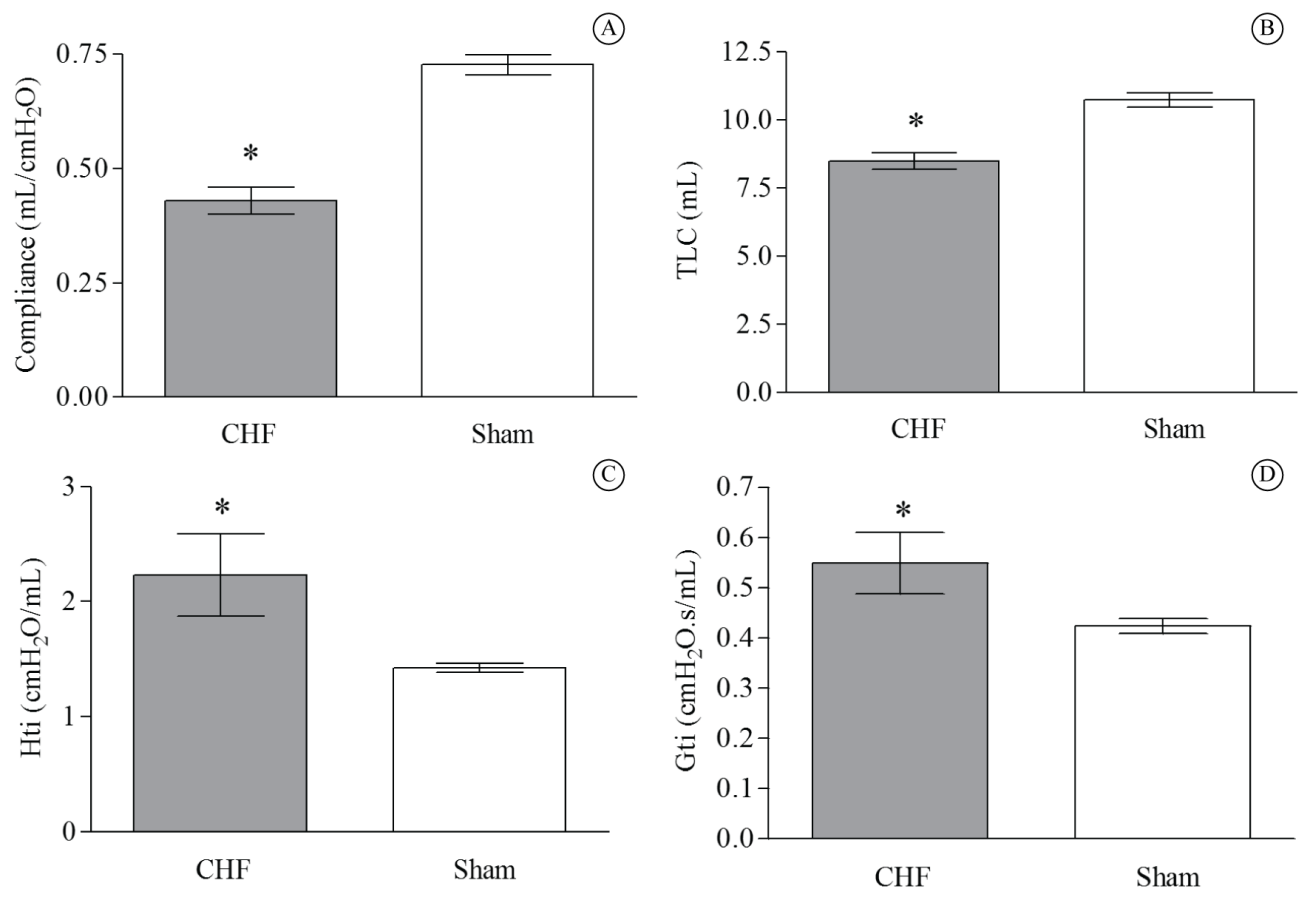
Respiratory mechanics variables in MI-induced CHF compared to sham-operated rats. CHF group (*n*=12) *vs* Sham group (*n*=15). (A) Respiratory system compliance (Crs) *P=0.0001; (B) Total lung capacity (TLC) *P=0.008; (C) Tissue elastance (Hti) *P=0.01; (D) Tissue resistance (Gti) *P=0.02. Values are means±SD.

The CHF group showed a decrease in Crs (*P*=0.0001) and TLC (*P*=0.008) values compared to the Sham rats (Figure 2A and 2B). Moreover, the CHF rats had higher Hti (*P=*0.01) and Gti (*P*=0.02) values than the Sham rats (Figure 2C and 2D).

### Correlations

**Figure 3:**
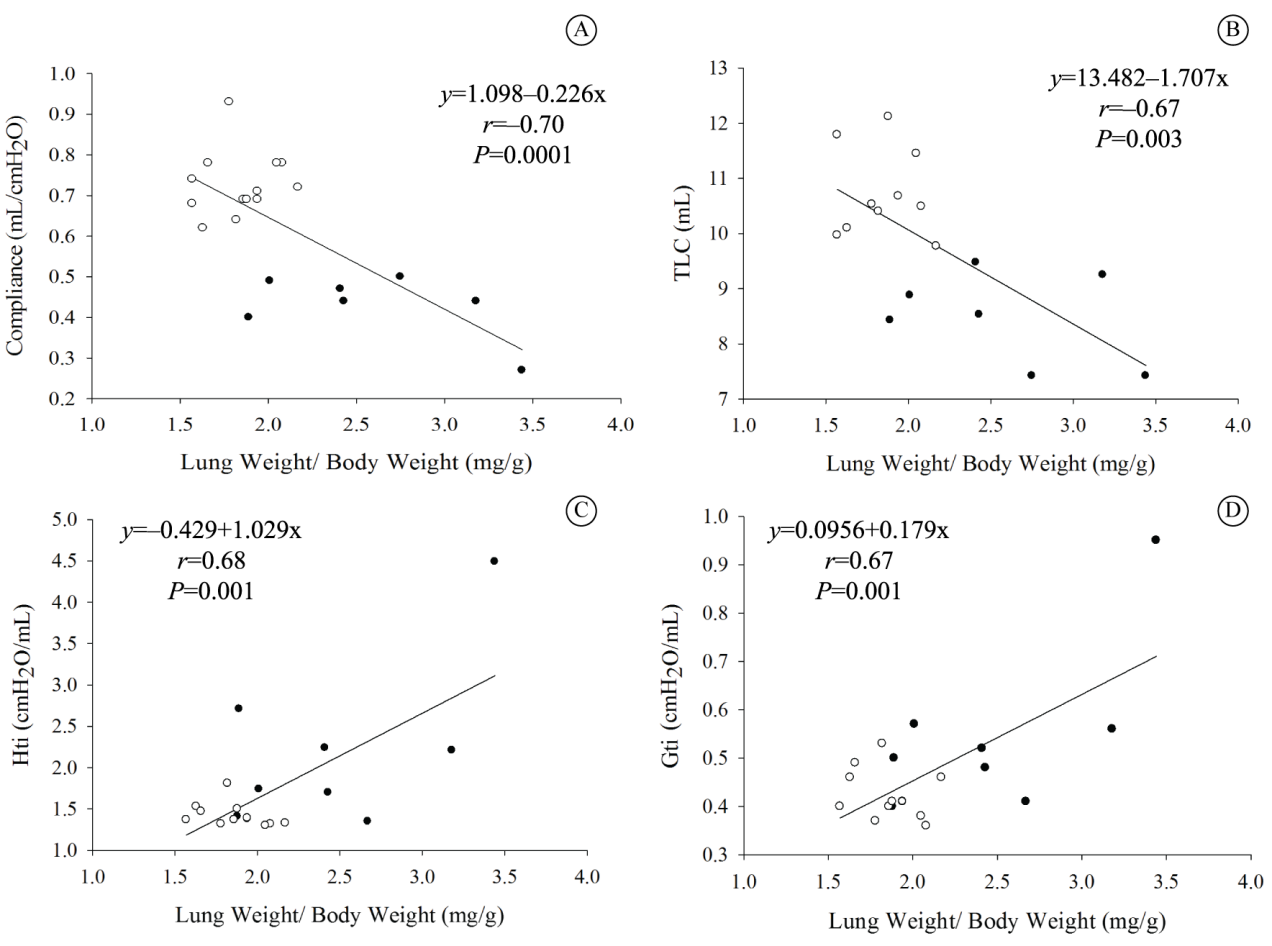
Correlations between pulmonary mechanical parameters and lung-to-body weight (LW∕BW) in Sham (○) rats and in the presence of CHF (●). (A) Respiratory system compliance (Crs) and (LW∕BW), CHF (*n*=7), and Sham (*n*=13); (B) Total lung capacity (TLC), CHF (*n*=7) and Sham (*n*=10); (C) Tissue elastance (Hti), CHF (*n*=8) and Sham (*n*=12); (D) Tissue resistance (Gti), CHF (*n*=8) and Sham (*n*=12). The slope represents linear regression.

**Figure 4:**
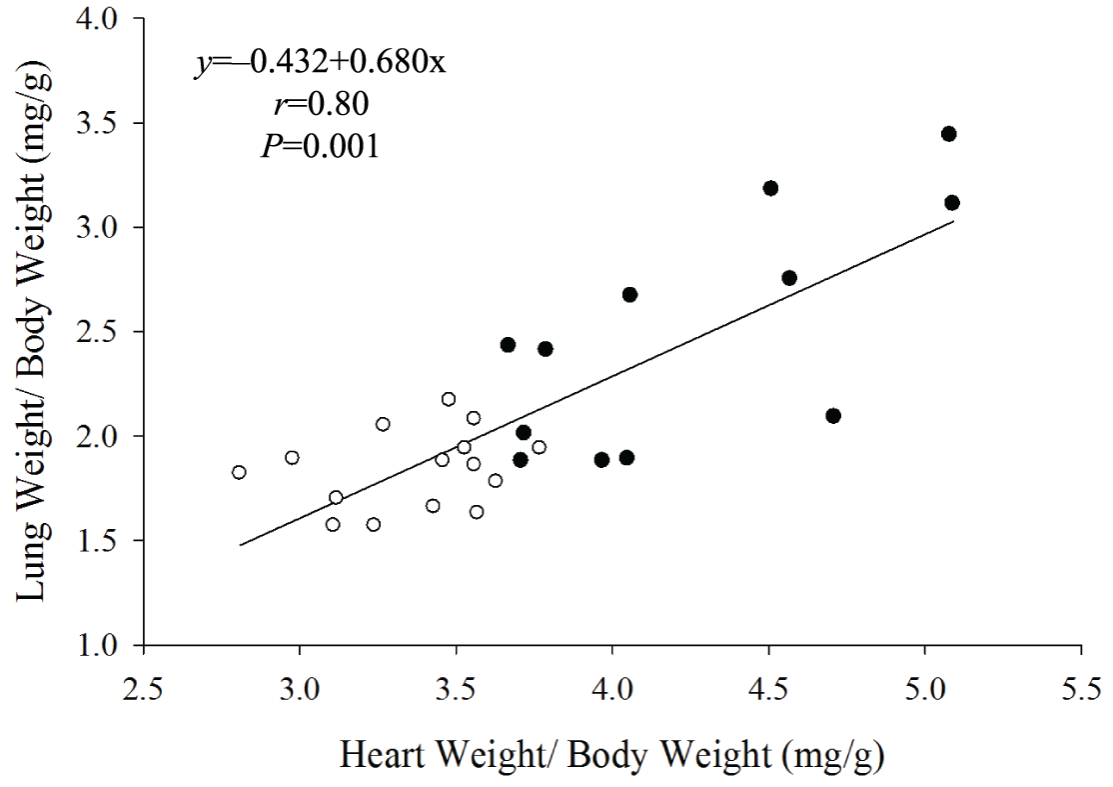
Correlation between lung-to-body weight (LW∕BW) and heart-to-body-weight (HW∕BW) in Sham rats (○, *n*=12) and in the presence of CHF (●, *n*=15). The slope represents linear regression.

A significant negative correlation was found between the Crs and TLC and the LW/BW ratios (Figure 3A and 3B), while Gti and Hti correlated positively with the LW/BW ratio (Figure 3C and 3D). In addition, there was a strong positive correlation between HW/BW and the LW/BW ratio (Figure 4).

## Discussion

The CHF rats showed increases in LVEDP and a reduction in LVSP and in + dP/dt_max_ and - dP/dt_max_, as well as increases in pulmonary edema and heart weight. We propose, therefore, that a chronic model of heart failure affects hemodynamic parameters and respiratory mechanics together, as demonstrated by the correlations between these variables in the experimental model of CHF.

Our data can be related to previous studies demonstrating that the left coronary artery ligature technique that is performed to induce an animal model of CHF produces marked left ventricular dysfunction that is directly related to the size of the infarcted area and simulates the most common cause of CHF^15^ ^,^ ^18^ ^,^ ^24^ ^,^ ^25^. Specifically, Nunes et al.^26^ observed that the average size of the infarcted area was around 34% of the total left ventricle area in the CHF groups, which produced a significant increase in LVEDP values (~36 mmHg). In accordance, here we observed similar LVEDP values, as well as other markers of CHF severity, such as decrements in the global LV contractility (+ dP/dt_max_) and relaxation (-dP/dt_max_) indices, as well as reduced left ventricular systolic pressure and heart rate. These alterations in hemodynamic parameters are closely related to decreases in inotropic and chronotropic function. In our study, significant alterations associated with cardiac remodeling and function were observed. Together, these data characterize the development of marked cardiac dysfunction in the CHF group. In fact, these hemodynamic alterations observed in the present study might induce structural and functional changes, which in turn may impair functional capacity as observed in the literature^27^.

Gehlbach and Geppert^8^ observed lower Crs values in heart failure patients, but the mechanisms involved with this condition were not fully elucidated. In this context, the changes in respiratory mechanics were found immediately after MI in an experimental model, which were followed by alterations in pulmonary resistive, elastic, and viscous-elastic components 2h after MI^13^. A previous study in rats showed that CHF changes the mechanical properties of the respiratory system^28^. These data corroborate our results, since we showed a reduction in Crs and TLC in the CHF group, characterizing the presence of significant alterations in pulmonary distensibility.

Another remarkable point to discuss is the increased LW/BW ratio observed in the CHF group, which is indicative of fluid accumulation and congestion in the lungs^18^. Together, these findings lead us to hypothesize that this model particularly affects the lung periphery, whereas no difference was found in the Rrs values between groups, which shows that the central airway is probably not affected. On the other hand, decreases in the Crs values are indicative of a more distal site of action, leading to a reduction in lung volume^9^ ^,^ ^10^. Bates and Irvin^29^ suggested that reductions in compliance reflect only the lung periphery, particularly airway closure.

Furthermore, the results reported herein regarding respiratory mechanical properties, such as Gti and Hti, corroborate the above-mentioned data, since there was an increased in Gti values but Rrs was unaltered, which most likely represents changes in the parenchyma or very small airways. Furthermore, chronic increases in Hti would be expected to reflect changes in the intrinsic properties of the lung parenchyma^9^ ^,^ ^10^. A reduction in pulmonary volume may produce increases in Gti and Hti that are wholly related to alterations in the mechanical properties of pulmonary tissue^21^. In addition, previous studies^4^ ^,^ ^30^ have demonstrated that increased airway resistance in the periphery of the lungs and reduced progressive compliance^13^ seem to be effects of pulmonary edema.

Pulmonary edema in CHF leads to modifications in the mechanical properties of the airways and pulmonary tissue due a chronic rise in microvascular pulmonary pressure^30^. These changes are preceded by interstitial edema, which is characterized by an accumulation of extravascular fluid, contributing to diminished pulmonary expansion^9^. In accordance, we presented here a close link between respiratory mechanical parameters and pulmonary edema.

It is important to note that the constant-phase model described by Hantos et al.^31^ identifies how an experimental intervention affects the size of the airways, the closure of alveolar units, and the regional heterogeneity of lung parenchyma^9^. In our study, we found increases in both Hti and Gti in the CHF group, which demonstrates the impact of a reduced expansion in the pulmonary tissue beyond increases in tissue resistance.

Interestingly, we observed a positive correlation between heart weight and pulmonary edema. In this regard, it has been widely reported that when LV dysfunction develops, the lung circulation and distal airway spaces become susceptible to untoward hemodynamic backward effects caused by elevated LVEDP and pulmonary capillary stasis^7^ ^,^ ^8^ ^,^ ^30^. These aspects are usually related to chronic state of compensated congestive heart failure, demonstrating that the hypertrophy commonly reported with CHF in this model^18^ ^,^ ^24^ ^,^ ^30^ worsens the pulmonary function, leading to fluid accumulation.

## Conclusions

Our data suggests that lung parenchymal mechanical dysfunction plays a pivotal role in respiratory pathophysiology of the CHF syndrome. Moreover, cardiovascular parameters are also modulated by these changes, supporting the idea that alterations in both systems are closely linked in CHF rats.
